# A whole-brain structural connectivity signature in adult Niemann–Pick disease type C

**DOI:** 10.1093/braincomms/fcaf426

**Published:** 2025-10-31

**Authors:** Thomas C Reilly, Maria A Di Biase, Sina Mansour, Caio Seguin, Vanessa L Cropley, Dennis Velakoulis, Andrew Zalesky, Christos Pantelis, Mark Walterfang

**Affiliations:** Systems Lab, Department of Psychiatry, The University of Melbourne, Melbourne, VIC 3010, Australia; The Neuropsychiatry Centre, The Royal Melbourne Hospital, Melbourne, VIC 3050, Australia; Systems Lab, Department of Psychiatry, The University of Melbourne, Melbourne, VIC 3010, Australia; Stem Cell Disease Modelling Lab, Department of Anatomy and Physiology, The University of Melbourne, Melbourne, VIC 3010, Australia; Department of Psychiatry, Brigham and Women's Hospital and Harvard Medical School, Boston, MA 02115, USA; Systems Lab, Department of Psychiatry, The University of Melbourne, Melbourne, VIC 3010, Australia; Centre for Sleep & Cognition and Centre for Translational Magnetic Resonance Research, Yong Loo Lin School of Medicine, National University of Singapore, Singapore 117597, Singapore; Systems Lab, Department of Psychiatry, The University of Melbourne, Melbourne, VIC 3010, Australia; Department of Psychiatry, The University of Melbourne, Melbourne, VIC 3010, Australia; The Neuropsychiatry Centre, The Royal Melbourne Hospital, Melbourne, VIC 3050, Australia; Department of Psychiatry, The University of Melbourne, Melbourne, VIC 3010, Australia; Systems Lab, Department of Psychiatry, The University of Melbourne, Melbourne, VIC 3010, Australia; Department of Biomedical Engineering, Melbourne School of Engineering, The University of Melbourne, Melbourne, VIC 3010, Australia; Department of Psychiatry, The University of Melbourne, Melbourne, VIC 3010, Australia; Monash Institute of Pharmaceutical Sciences (MIPS), Monash University, Parkville, Melbourne, VIC 3052, Australia; The Neuropsychiatry Centre, The Royal Melbourne Hospital, Melbourne, VIC 3050, Australia; Department of Psychiatry, The University of Melbourne, Melbourne, VIC 3010, Australia; Florey Institute of Neuroscience and Mental Health, Melbourne, VIC 3052, Australia

**Keywords:** Niemann-Pick, connectivity, neurodegeneration, networks, genetics

## Abstract

Niemann–Pick disease type C (NPC) is a rare genetic lysosomal storage disorder with a clinically heterogeneous phenotype that primarily affects the brain, liver and spleen. Most cases of NPC are diagnosed in childhood, but a subset of patients who are diagnosed in adulthood present with psychiatric symptoms and are initially diagnosed as schizophrenia or a mood disorder. Neuroimaging studies in NPC show a predilection for neurodegeneration in the subcortical nuclei, the cerebellum and subcortical white matter. We aimed to explore how adult NPC affects the connectivity of networks and hypothesized a state of widespread disconnectivity, particularly in subcortical areas. This cross-sectional neuroimaging study used diffusion-weighted magnetic resonance imaging to perform whole-brain tractography in 9 adult patients with NPC and 70 matched healthy controls. Connections between 84 unique brain regions were modelled with streamlines and weighted according to fibre bundle capacity. Statistical testing by each connection allowed the identification of significantly affected networks and regions in NPC. We observed diffusive disconnectivity in NPC primarily affecting left frontotemporal and subcortical networks. Globally and regionally, NPC showed reductions in the strength and number of affected connections, particularly in the thalamus and dorsal striatum. We show that left frontotemporal and subcortical networks are markedly affected in NPC. These findings may facilitate early diagnostic differentiation, monitoring and prognostication and may represent the neural correlates of neuropsychiatric symptoms in NPC.

## Teaser

Reilly *et al*. model brain structural connectivity in Niemann–Pick disease type C using diffusion-weighted MRI. They demonstrate the extent and distribution of connection loss that particularly affects the frontal, temporal and subcortical brain regions. This foundational work may contribute to improving diagnosis, disease monitoring and prognostication.

## Graphical Abstract

**Graphical Abstract:**
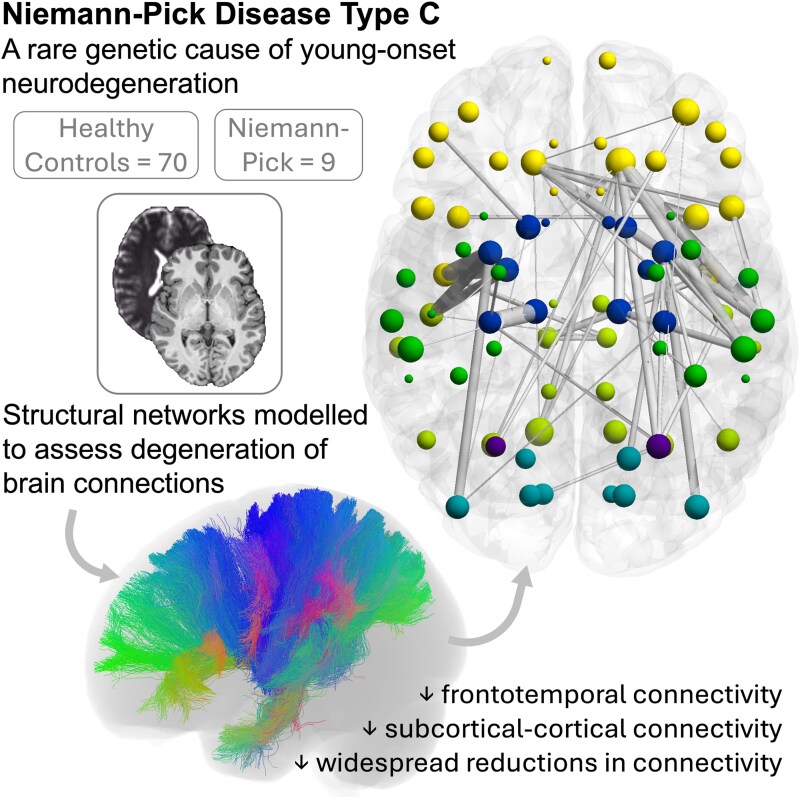

## Introduction

Niemann–Pick disease type C (NPC) is a rare, autosomal recessive disorder caused by mutations in the NPC1 or NPC2 genes, which disrupts cholesterol and lipid trafficking, particularly in the liver, spleen and brain.^1-6^ Neuronal glycosphingolipid and cholesterol accumulation lead to ectopic dendritogenesis, neuronal body swelling and cytoskeletal dysfunction.^7^ Axonal structure is affected by altered myelination, meganeurite formation and axonal spheroids.^8^ Accumulative neuronal pathology causes neurodegeneration, which includes the formation of neurofibrillary tangles, tau accumulation and neuroinflammation.^9,10^

Structural magnetic resonance imaging (MRI) studies have shown widespread grey and white matter atrophy, particularly in the thalamus, basal ganglia, cerebellum and brainstem.^11,12^ Longitudinal studies confirm that structural changes are progressive^11,13^ and correlate with areas of active neuroinflammation.^10^ Diffusion-weighted MRI (dMRI) studies show altered white matter in large central white tracts such as the corona radiata, the internal capsule, the corpus callosum and the cingulum bundle, implicating widespread structural disconnectivity as underlying some of the key symptoms of the disease.^11-13^

Using dMRI, whole-brain tractography, followed by network analysis, can interrogate structural connectivity alterations in disorders such as NPC. This approach builds upon the established literature interrogating white matter tracts by providing a whole-brain, system-level approach to detecting changes in brain wiring. Network analysis involves reconstructing a representation of white matter architecture (i.e. connections) between all pairs of brain regions (i.e. nodes) comprising a grey matter parcellation. Advances in structural tractography methods have improved the biological reliability of these connections, including quantifying connectional capacity through fibre bundle capacity (FBC).^14,15^ Subsequent connectivity analysis enables between-group comparisons of each brain connection using network-based statistic (NBS).^16^ One previous study has shown widespread decreases in connectional fractional anisotropy (FA) and altered network topology in NPC.^17^ More work is thus needed to build on these findings and characterise how NPC affects the capacity for connection between brain regions.

Clinically, adult-onset NPC can present with neuropsychiatric symptoms such as psychosis, mood disorder and cognitive impairment that progresses to dementia.^18^ Misdiagnosis of a primary psychiatric condition delays diagnosis and misses opportunities to slow neurodegeneration with illness-modifying treatment. dMRI tractography has revealed progressive disconnectivity in neurodegenerative conditions such as Huntington’s Disease^19^ and Alzheimer’s Disease,^20^ suggesting that an MRI-derived signature of NPC could similarly aid early diagnostic differentiation and prompt definitive genetic testing. The extent and distribution of structural disconnectivity may support clinical monitoring of disease progression, treatment response and prognosis. This exploratory work may also clarify the neural correlates of neuropsychiatric symptoms such as psychosis and dementia.

## Materials and methods

### Participants and design

This cross-sectional study leveraged existing cohorts of 9 participants with adult NPC (NPC)^10^ and 70 healthy controls (HC) for 79 total participants.^21,22^ Participants were matched for age and sex. Group demographics are presented in Table 1.

**Table 1:** Group demographics

		Sex				Age					
Group	*N*	M:F	χ²	df	*P*	Mean	SD	SE	Student’s *t* statistic	df	*P*
HC	70	43:27	0.955	1	0.329	37.8	10.9	1.31	1.43	77	0.157
NPC	9	4:5				32.2	11.6	3.86			

The cohort of patients with adult NPC is associated with a specialist neuropsychiatry clinic at The Royal Melbourne Hospital. Diagnosis was confirmed with genotyping, and all demonstrated disease-causing mutations in NPC1. Shared exclusion criteria for the NPC and HC cohorts included: history of significant head injury, current pregnancy or breastfeeding, presence of MRI contraindications, impaired thyroid function, diabetes, daily use of steroidal or non-steroidal anti-inflammatory, immunosuppressive, corticoid or glucocorticoid drugs continuously for more than 1 week within the last 6 months and generalized inflammatory condition or disease. This study was approved by the Austin Health and Melbourne Health ethics committee, and participants provided written informed consent. The study was conducted in accordance with the Declaration of Helsinki (1964).

### MRI acquisition

T_1_-weighted and dMRI scans were acquired on a 3T Siemens (Munich, Germany) Trio at Murdoch Children’s Research Institute, Royal Children’s Hospital, Australia. T_1_-weighted acquisition parameters were 3D spoiled gradient T_1_-weighting, echo time = 3 ms, repetition time = 14 ms and 256 contiguous whole brain slices with 1 × 1 × 1 mm voxels. For diffusion-weighted acquisition, an echo planar imaging sequence was used. 60 diffusion-weighted volumes and 10 diffusion-unweighted, b0, volumes were acquired with the following parameters: b-value = 3000 s/mm^2^, slice thickness 2 mm, image matrix 104 × 104, in-plane voxel resolution 2 × 2 mm, field of view 208 × 208, repetition time 7.750 ms, echo time 112 ms, flip angle 90°. Imaging was repeated if there was gross movement artefact as determined by the radiologist.

### MRI pre-processing

Pre-processing of MRI images was primarily carried out through MRtrix3^23^ and FSL^24^ software. The raw diffusion-weighted images were initially denoised by subtracting the estimated thermal noise residuals.^25^ The resulting images were then processed to remove Gibbs ringing artefact.^26^ Due to the lack of reverse phase-encoded images in the acquisition, Synb0-Disco was used to facilitate the correction of susceptibility-induced distortions.^27,28^ Synb0-Disco synthesises an undistorted image from the T_1_-weighted scan to produce a new image that geometrically matches the T_1_ image with diffusion image contrast. The outputs were then fed into FSL’s eddy current-induced distortions and movement correction algorithm, ‘eddy’.^29^ Finally, the images were corrected for low-frequency intensity non-uniformity using the N4ITK algorithm.^30^ FSL’s ‘DTIFit’ algorithm was used to create maps of white matter diffusion properties, such as FA, based on the diffusion tensor model at each voxel.

Structural T_1_ MRI images were processed using FSL’s ‘fsl_anat’ script. Images were corrected for bias field inhomogeneity using FSL’s ‘FAST’ algorithm.^31^ Brain extraction was completed using FSL’s ‘brain extraction tool’ (BET).^32^ Structural T_1_ images were then registered to native diffusion space in a two-step process: first, the affine transformation was computed using FSL’s ‘FLIRT’,^32^ and next, the linear transformation was applied using MRtrix3’s ‘mrtransform’.^23^

### White matter tractography

The fibre orientation distribution was estimated from the diffusion image using constrained spherical deconvolution (CSD) in MRtrix3. The tissue-specific response functions for white matter, grey matter and cerebrospinal fluid were estimated from each image using an unsupervised method.^33^ Single-shell, 3-tissue CSD was then used to generate fibre orientation distributions (FODs)^34^ using MRtrix3Tissue,^35^ a fork of MRtrix3.^23^ The FODs were then normalized to correct for intensity inhomogeneities.^36^

Anatomically constrained tractography generated streamlines based on the computed white matter FODs.^37^ This method uses the T_1_ image segmented into white matter and grey matter components to identify the grey matter-white matter interface. Probabilistic tractography using the iFOD2 algorithm delineated streamlines between grey matter regions.^38^ 25 million streamlines were seeded randomly from the grey matter-white matter interface. Only streamlines that interconnected grey matter were deemed valid. The white matter FODs determined the course of the streamlines, where streamlines are more likely to progress along a path where the FOD amplitudes are large.

### Cortical parcellation

Cortical parcellation was initially conducted through the FreeSurfer automated ‘recon-all’ algorithm,^39^ which was used to parcellate the pre-processed T_1_ image according to the Desikan–Killiany atlas. This parcellation was then applied to the volumetric image, along with the addition of subcortical nuclei, brainstem and cerebellum parcellations, resulting in a total of 84 unique brain regions.^40^ These parcellated images were then transformed into native diffusion space by applying the inverse linear transformation matrix using MRtrix3’s ‘mrtransform’ command.

### Network construction

Parcellations and streamlines were combined to form a connectivity matrix for each subject, where each row and column of the matrix represents brain regions, and each element denotes the measure of connectivity between two regions. The connectivity matrices were then weighted according to four measures of connectivity strength: (1) estimated FBC, (2) streamline count, (3) mean streamline length and (4) tract-averaged FA. FBC was the principal measure of connectivity used in this analysis, as well as the between-groups statistical comparison. FBC was inferred from a connection's total intra-axonal cross-sectional area using MRtrix3’s ‘SIFT2’ algorithm.^14,15^ The streamline count is simply the number of generated streamlines connecting two regions. Supplementary Table 1 contains explanations of the definition and interpretation of each of the connectivity measures calculated. Global connectivity was calculated by summing or averaging the connectivity measures. Nodal connectivity was calculated by summing or averaging the connectivity measures at a specific brain region.

### Statistical testing for connectivity alterations

Global measures were compared between groups, and non-parametric two-tailed Mann–Whitney U-tests were used with a Bonferroni-adjusted *P-*value (*P* = 0.0125) to control for multiple comparisons across the four measures. Nodal FBC was calculated by summing the FBC of all connections to a region and then comparing this between groups. Rank biserial correlation coefficient was used to calculate the standardized extent and direction of the effect size of NPC per region due to the non-normal distribution of nodal connectivity weights. Statistical tests for connectivity per region were not performed to limit the chance of family-wise error and because this statistical inference is captured in the network analysis. Jamovi statistical software was used for between-groups analysis and calculating effect sizes.^41^

The NBS was used to localize differences in connectivity strength between NPC and HC groups using the FBC-weighted connectivity matrices as input. NBS tests for a difference using a two-sample test at each connection to identify network clusters that exceed an effect size threshold.^16^ 5000 permutations were used to generate the null distribution with a cluster-forming threshold of *P* < 0.05. Various effect size thresholds were then used to characterise the significantly altered sub-networks in NPC.

### Visualizations

**Figure 1:**
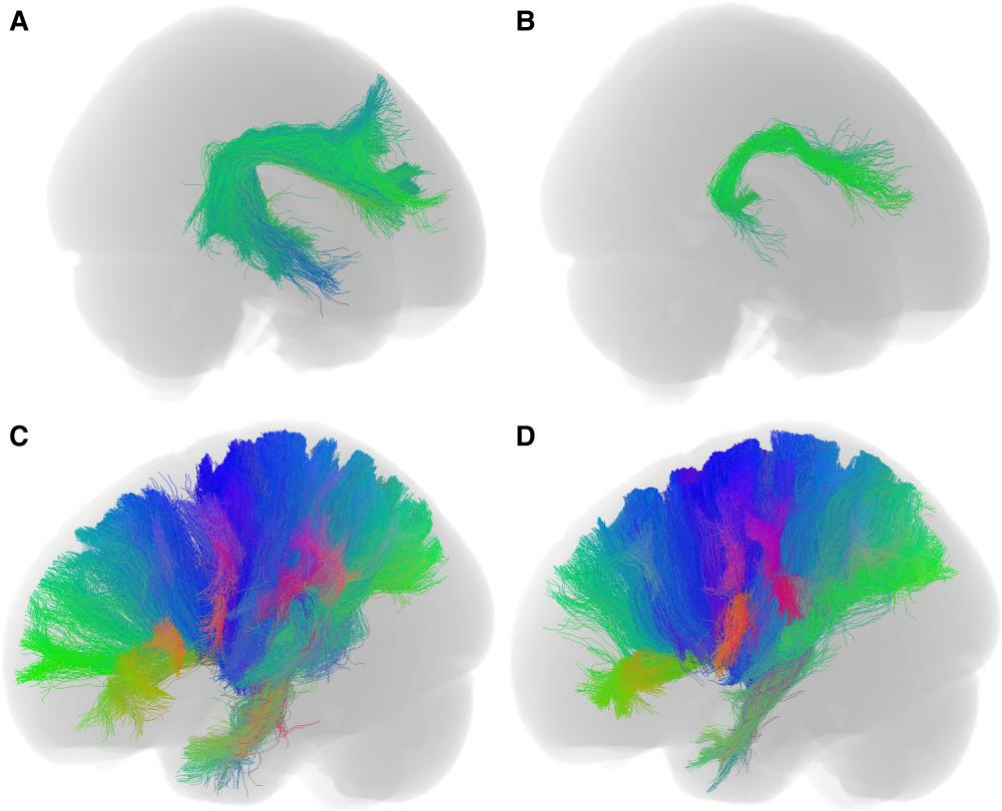
**Representative tracts.** (**A** and **B**) show the left arcuate fasciculus of HC and NPC representatives, respectively. (**C** and **D**) show the left corticothalamic tracts of HC and NPC representatives, respectively.

**Figure 2:**
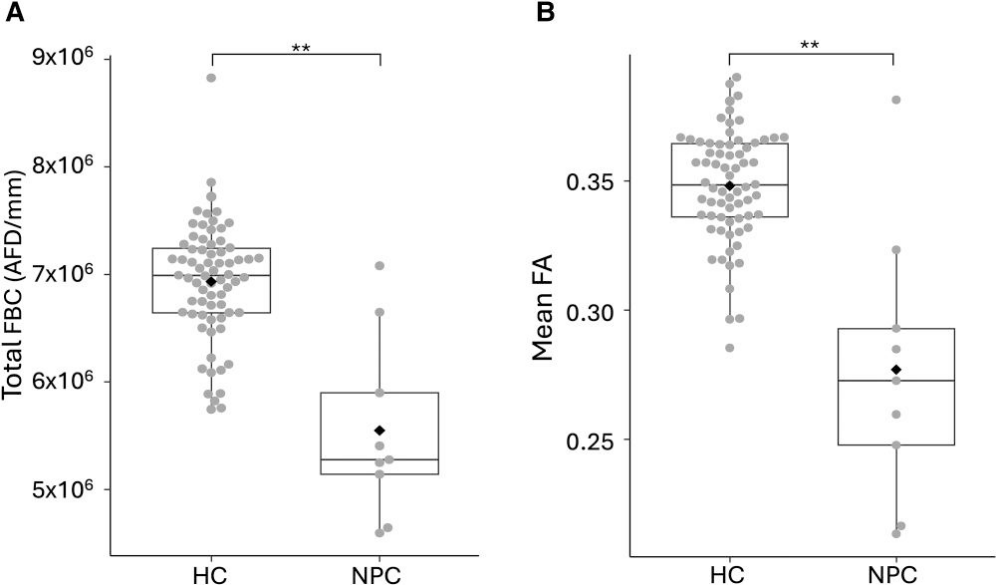
**Disruptions in global FBC and FA in NPC.** Boxplots represent the total FBC (**A**), the global mean FA (**B**) for NPC (*n* = 9) and HC (*n* = 70). Each data point represents a subject, black diamonds represent means, centre bars represent medians, boxes represent 25th and 75th percentiles, and whiskers represent 10th and 90th percentiles. **P < 0.001 significance level determined by Mann–Whitney U-tests. FBC = fibre bundle capacity; AFD = apparent fibre density; FA = fractional anisotropy.

**Figure 3:**
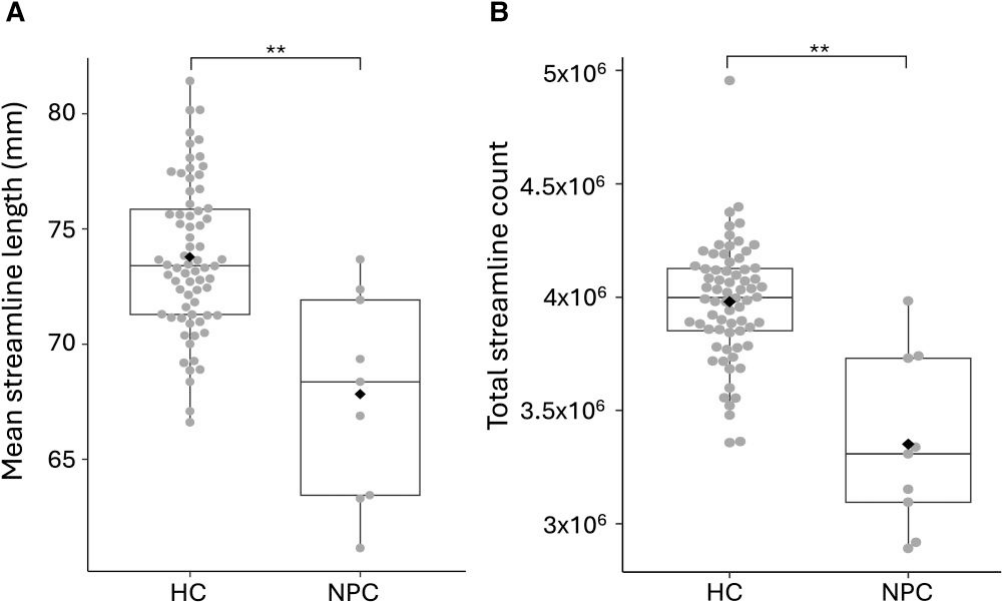
**Disruptions in global connection length and streamline count in NPC.** Boxplots represent the average connection length (**A**) and total streamline count (**B**) for NPC (*n* =9) and HC (*n* = 70). Each data point represents a subject, black diamonds represent means, centre bars represent medians, boxes represent 25th and 75th percentiles, and whiskers represent 10th and 90th percentiles. **P < 0.001 significance level determined by Mann–Whitney U-tests.

**Figure 4:**
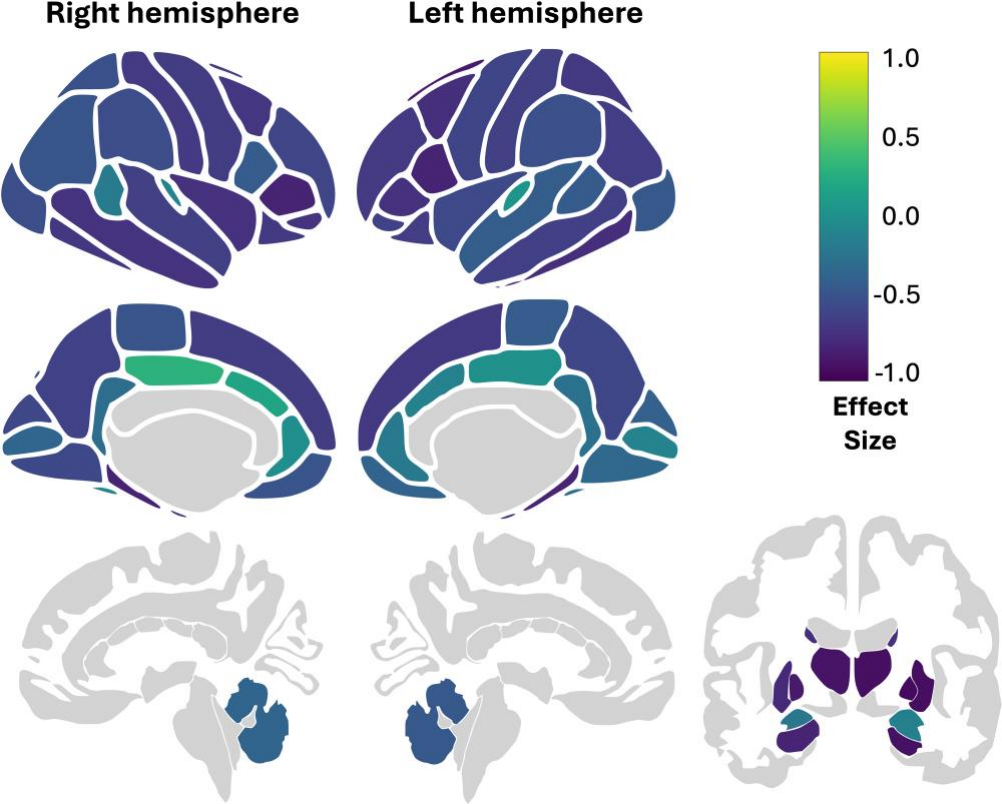
**Effect size of alterations in fibre bundle capacity of cortical and subcortical brain regions.** Effect sizes of NPC (*n* = 9) versus HC (*n* = 70) for each region calculated using rank biserial correlation coefficient. Purple indicates reductions of FBC in NPC; yellow indicates increases in FBC in NPC.

**Figure 5:**
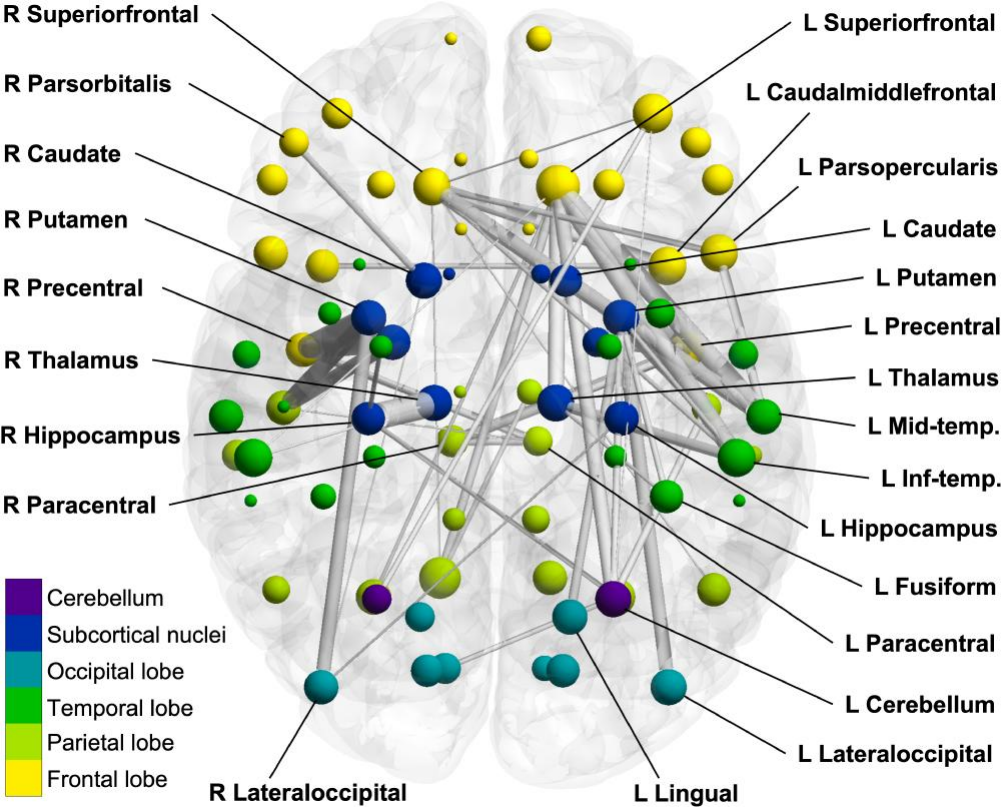
**Visualization of NBS results.** Each node is shown as a sphere. Only nodes present at threshold level of 3 shown (70 in total). Node size indicates the degree, or number of significant connections. Nodes are coloured according to lobes for visualization. Only edges with a *T*-statistic greater than 4 are shown for visualization. Edge thickness indicates the corresponding *T*-statistic. L = Left; R = Right; Mid-temp = Middle Temporal; Inf-Temp = Inferior Temporal.

Tract segmentation and visualization (Fig. 1) were performed using DiPY RecoBundles.^42^ Boxplots (Figs 2 and 3) were generated using RStudio.^43^ Brain segmentations (Fig. 4) were generated using ‘ggseg’ for Python (https://github.com/ggseg/python-ggseg). Visualization of NBS results (Fig. 5) was generated using BrainNet Viewer.^44^

## Results

### Clinical and demographic details

The cohort consisted of 70 HCs (43 males, mean age 37.8 ± 10.9 years) and 9 participants with NPC (5 females, mean age 32.2 ± 11.6 years). HC and NPC groups were matched for age (*P* = 0.157) and sex (*P* = 0.329) (see Table 1). Clinical details for the NPC group, including age of diagnosis, duration of illness, illness severity and the presence of psychotic symptoms, can be found in Supplementary Table 2.

### Connectivity alterations in NPC

Visual inspection of tract-segmented streamlines showed marked deficits in the NPC group. Figure 1 shows group exemplars of the left arcuate fasciculus tract and the left corticothalamic projections. Global measures in NPC displayed significantly lower total FBC, mean FA, streamline count and average streamline length (see Figs 2 and 3 and Table 2). The distributions of global connectivity measures are presented in Supplementary Table 3. The results were particularly striking in the FBC and mean FA measures, suggesting that axonal integrity and communicative capacity are reduced in NPC. Global measures are recapitulated at the nodal scale, where widely distributed decreases in FBC were observed, with the thalamus and basal ganglia being the most affected and frontal and temporal regions with high rank biserial correlation (see Fig. 4). These findings at the global and nodal levels align with both previous dMRI and pathological studies in NPC, which demonstrate the extent of damaged neuronal wiring in NPC.^7,11^

**Table 2:** Connectivity measures at a global level

	Statistic		*P*	Diff. of means	% change	Effect size^a^
Total FBC	Mann–Whitney U	64	< 0.001	−1.38 × 10^6^	−19.91	−0.797
Average streamline length	Mann–Whitney U	94	< 0.001	−5.956	−8.07	−0.702
Total streamline count	Mann–Whitney U	55	< 0.001	−6.3 × 10^5^	−15.83	−0.825
Mean FA	Mann–Whitney U	77	<0.001	−0.071	−20.4	−0.756

Diff. = Difference.

^a^Effect sizes calculated using rank biserial correlation.

Clinical characteristics of NPC were associated with global connectivity measures (Table 3). A longer duration of illness was associated with a lower global mean FA while controlling for the effects of age (Pearson’s *r* = 0.713, *P* = 0.047). Older age of diagnosis was also associated with lower global mean FA (Pearson’s *r* = −0.710, *P* = 0.048) as well as reduced average connectional length (Pearson’s *r* = −0.747, *P* = 0.033). These findings suggest that an older age of diagnosis and a longer duration of illness are associated with a greater loss of connectional integrity, particularly affecting longer connections. Importantly, while clinical associations with global connectivity showed strong *P*-values for a small cohort, no analyses survived Bonferroni correction (*P* < 0.00417). The presence of psychosis (*n* = 2) was associated with lower average connectional length (mean difference −7.21, SE 2.69, *P* = 0.031) (Table 4). While acknowledging the weakness of low numbers, this finding suggests that psychosis in NPC is associated with loss of longer connections. There were no sex differences in global connectivity metrics (Supplementary Table 4).

**Table 3:** Partial correlation of clinical characteristics and connectivity measures in NPC controlled for age

		Age Dx	DOI	Illness severity
Total FBC	Pearson's *r*	−0.369	0.621	−0.369
	*P*-value	0.368	0.1	0.369
Mean FA	Pearson's *r*	*−0*.*710*^a^	*0*.*713*^a^	−0.317
	*P*-value	0.048	0.047	0.444
Average length	Pearson's *r*	*−0*.*747*^a^	0.232	−0.285
	*P*-value	0.033	0.58	0.493
Total streamlines	Pearson's *r*	−0.182	0.689	−0.244
	*P*-value	0.667	0.059	0.561

Note: controlling for ‘age’. Bonferroni correction *P* < 0.00417.

^*a*^ *P*  *<* 0.05; **P  *<* 0.01; ***P  *<* 0.001.

**Table 4:** Comparison of psychotic versus non-psychotic NPC according to global connectivity

	Student’s *t*	df	*P*	Mean diff.	SE diff.	Cohen’s *d*
Total FBC	0.5441	7	0.603	387 372.3	712 009.7	0.4362
Mean FA	1.2778	7	0.242	0.052	0.0407	1.0246
Average length	2.6815	7	*0*.*031*^a^	−7.2079	2.6881	2.1499
Total streamline count	0.082	7	0.937	27 222.5	331 920.7	0.0658

Note: H_a_ μ_0_ ≠ μ_1_. Diff. = difference.

^a^Does not survive Bonferroni correction (*P* < 0.0125).

We next sought to interrogate whether disconnectivity in NPC manifested as a subnetwork of edges that may suggest a signature of impaired wiring in NPC. Edge-wise statistical testing of the FBC-weighted connections using the NBS with 290 edges and 70 nodes being at least three standard deviations below normal (‘Threshold 3’, *P* < 0.001; see Table 5). There was a subcortical and left frontotemporal disconnectivity pattern among the significantly widespread disconnection. The striatum (putamen and caudate), the thalamus, the superior frontal and caudal middle frontal gyri and the inferior temporal gyri were the most strongly involved. Table 5 shows the results of NBS testing. Supplementary Table 5 shows the list of edges with the highest test statistic, along with the associated FBC, streamline count, mean FA and edge length. Figure 5 shows the disconnectivity network in NPC. All nodes participating in the significant network are shown as spheres coloured by general lobar location. The size of each node represents its strength within the significant network. Edges (grey lines) with a *T*-statistic of greater than 4 are shown to facilitate better visualization of the most affected connections. The size of the edges reflects the *T*-statistic of each edge.

**Table 5:** Results of network-based statistic analysis^a^

Threshold	1	2	3	4
No. of networks	1	1	1	1
Lowest *P*-value	<0.001	<0.001	<0.001	<0.001
No. edges	2229	1000	290	51
No. nodes	84	84	70	38

^**a**^NBS performed testing NPC < HC hypothesis at four thresholds.

## Discussion

### Reduced network connectivity extent

Using whole-brain tractography, we demonstrated that the NPC brain is widely disconnected, with a predominance of subcortical networks and frontotemporal cortical networks. We demonstrate a strong overall effect of NPC on structural connectivity, despite the small sample size. Network-level analysis using FBC-weighted networks shows a dense and widely distributed reduction in connectivity. The summed FBC of regional connections has also decreased, emphasizing the distribution of disconnectivity. At the global level, total FBC, mean FA, total streamline count and mean connection length were significantly reduced (all *P* < 0.001). Only one other study has investigated connectivity in NPC, albeit using a different method of tractography production (3DSlicer) and a slightly older cohort (mean age 36.00).^17^ This work found reduced nodal degree (the number of connections at a brain region) in the left pars triangularis, the left calcarine region, the right inferior parietal cortex and the right anterior ventral thalamus.^17^

Our results show that the multifaceted neuronal pathology in NPC reduces the capacity for connection between brain regions. The known accumulation of gangliosides, other neurotoxic compounds and abnormal microglial activation contribute to the loss of axonal fibres, resulting in reduced FBC. The reductions in FA values in NPC connections also indicate a disruption in the connection microstructure. These findings are supported by previous work in NPC, which shows active inflammation in the white matter pathways.^10^

### Cortical–cortical disconnectivity

Edge-wise analysis suggests two patterns of disconnection: in the left hemispheric frontal-temporal-parietal association tracts and a background of widespread disconnection. This distribution indicates a susceptibility of large longitudinal tracts such as the arcuate fasciculus, superior longitudinal fasciculus or the cingulum bundle. Commissural connections involving the right superiofrontal and left-sided frontotemporal regions were also highly affected.

The lateralization of neuropathology in NPC has previously been noted, with FA being most reduced in the left cingulum.^17^ The lateralization of left-sided frontotemporal circuits suggests a susceptibility of the dominant-sided arcuate fasciculus. This may be due to an inherent disposition of the left frontotemporal circuitry to pathological mechanisms in NPC neurodegeneration. Alternatively, left-bias disconnectivity may be partly explained by the heterogeneity of lateralization of the arcuate fasciculus.^45^ The arcuate fasciculus is typically larger in the dominant hemisphere,^46^ and a highly myelinated tract with a larger surface area and higher cholesterol demands may be more susceptible to NPC pathology. There is also evidence of asymmetry in the microstructure of large white matter tracts,^47^ which may render the left frontotemporal tracts vulnerable to NPC pathophysiology.

Frontotemporal disconnectivity provides a basis for understanding how NPC can clinically resemble frontotemporal and other dementias.^48^ Altered diffusivity measures in large white matter tracts have been shown in behavioural variant FTD, including in the left superior longitudinal fasciculus.^49^ Hypo and hyper-connectivity in fronto-striatal and fronto-thalamic connections have also been observed in behavioural variant FTD.^50^ Our results suggest that the clinical resemblance between NPC and FTD may stem from overlapping patterns of frontotemporal disconnectivity.

Mesial temporal lobe and hippocampal white matter dysconnectivity is a consistent finding in Alzheimer’s Dementia (Ad), present preclinically and progressing to the structurally connected frontal and parietal regions.^51^ Structural network alterations in Ad also appear to be driven by the cumulative spread of tau, a process similarly observed in NPC.^52,53^ Our results suggest that the pathological accumulation of tau may also drive structural network changes in NPC that resemble those in Ad.

### Disconnectivity and clinical features

We show here that older age of diagnosis and duration of illness are associated with deficits in connectional microstructure as measured by FA. An older age of diagnosis is also associated with a reduced connectional length. Prolonged periods of untreated illness, potentially due to diagnostic delays, may result in greater degeneration of structural connections. This preliminary finding, although limited by sample size, suggests that structural connectivity may be a valuable tool for monitoring disease progression and treatment response.

It has been proposed that psychotic symptoms in NPC, often mistaken for schizophrenia, may emerge from a state of widespread dysconnectivity.^18^ Subtle, widespread and frontotemporal white matter disconnectivity has been observed in schizophrenia, including in first-episode cohorts.^54-56^ Thus, our findings suggest that the state of widespread and frontotemporal disconnectivity in NPC may represent a connectivity correlate of psychotic symptoms.^17^ Our analysis also revealed that psychotic patients with NPC tended to have shorter connection lengths than non-psychotic patients with NPC. This intriguing finding, though limited by group sizes, has not been directly reported in the literature on structural connectivity in schizophrenia. These observations prompt the hypothesis that the loss of long white matter connections is a marker of psychosis in neurodegenerative pathology or primary schizophrenia.

### Subcortical disconnectivity

It is well established that the thalamus and the basal ganglia are vulnerable to degeneration in NPC.^13^ Here, we show the significant loss of thalamic projections to the frontotemporal regions. Mouse studies indicate the importance of thalamocortical connections and the abnormal glial environment in NPC.^57^ Thalamic disconnectivity and the consequent loss of ability to integrate complex information possibly also explain the susceptibility to psychotic symptoms and executive dysfunction seen in NPC.

Putaminal and pallidal connections showed significantly reduced FBC, particularly in the motor areas, including the precentral and postcentral gyrus and the cerebellar cortex. These connections also showed reduced streamline count and reduced FA. This finding accords with previous research showing that the primary motor networks and projections are affected by the consequences of lipid accumulation in NPC.^12,13^

### Limitations

This study is limited by the low numbers of the NPC group, which is an inherent weakness of all studies in ultrarare disorders, and is perhaps more pronounced in neuroimaging studies. As mentioned above, some connections were flagged as significantly reduced with a very low number of streamline connections. Even in a healthy brain, connections with low streamline counts must be interpreted with lower confidence. Notably, many of the commissural connections flagged in the edge-wise analysis contained fewer than 100 streamlines in the NPC group, which, although reduced compared to the HC, does imply a higher level of uncertainty about their validity.

## Conclusion

This study interrogates the neurodegeneration of white matter connectivity in NPC. Strong findings in the thalamus, striatum and frontotemporal circuits are commensurate with understanding the regions affected by neurodegeneration in NPC. This study also highlights how the multifaceted neuronal pathology translates to impaired meta-states of disconnection in NPC. Key subcortical regions show dysfunction in the extent and organization of connections, while frontotemporal regions show the effects of large white matter bundles. These findings contribute to the formation of a neuroimaging and disconnectivity signature of NPC, potentially relevant to other lysosomal storage disorders. There is potential for future use in diagnostic differentiation, especially in the cohort initially misdiagnosed as a primary psychiatric condition. This work may also translate to better prognostication and treatment monitoring in NPC. Although the translational potential of these findings remains hypothetical, this exploratory study provides foundational insights to be validated in longitudinal studies with larger datasets.

## Supplementary Material

fcaf426_Supplementary_Data

## Data Availability

All processing was conducted using existing code from the software packages listed in the Methods section. Example code for imaging processing steps can be found in the Supplementary material or on the software package websites. Please contact the corresponding author for further requests.
