# Supplementary material for: A whole-brain structural connectivity signature in adult Niemann–Pick disease type C
Source: Brain Commun. 2025 Oct 31;7(6):fcaf426. doi: 10.1093/braincomms/fcaf426 (PMC12631124; doi:10.1093/braincomms/fcaf426)
Supplement: fcaf426_Supplementary_Data [file fcaf426_supplementary_data.docx]

**Supplementary Material**

| **Supplementary Table 1. Definition and interpretation of connectivity measures** | | |
| --- | --- | --- |
|  | **Definition** | **Interpretation** |
| Fibre bundle capacity | The sum of all connection weights in the brain, where connection weightings are determined by estimated fibre bundle capacity. | A measure of the extent of connectivity weighted by the estimated fibre bundle capacity. Fibre bundle capacity is an approximation of the cross-sectional area of a bundle and is modulated by white matter microstructure. |
| Streamline count | The number of streamlines generated that connect one brain region to another. This is calculated during structural connectome production. | A measure of extent of connectivity where larger and more robust neuronal pathways allow greater number of streamlines connecting two brain regions. Streamline count is a virtual representation of connectivity that is not modulated by white matter microstructure. |
| Streamline length | The average length of streamlines connecting two brain regions. This is calculated during structural connectome production | Shorter connection lengths can indicate alterations of white matter tracts that divert or prematurely complete the generated streamlines. Shorter pathways may also indicate global atrophy. |
| Mean FA | The average Fractional Anisotropy value of all connections between brain regions. This is calculated by sampling the FA values underlying streamlines. | Mean FA is an indicator of disruption of axonal integrity during processes such as demyelination. |

| **Supplementary Table 2. Clinical Characteristics of NPC group** | | | | | |
| --- | --- | --- | --- | --- | --- |
| **Participant** | **Age** | **Age of Dx** | **DOI** | **Illness Severity** | **Psychosis Status** |
| NPC1 | 52 | 45 | 12 | 6 | 1 |
| NPC2 | 41 | 34 | 13 | 12 | 1 |
| NPC3 | 38 | 37 | 12 | 11 | 3 |
| NPC4 | 23 | 20 | 5 | 10 | 2 |
| NPC5 | 20 | 16 | 7 | 14 | 1 |
| NPC6 | 18 | 9 | 10 | 5 | 1 |
| NPC7 | 27 | 18 | 11 | 16 | 1 |
| NPC8 | 29 | 20 | 8 | 7 | 1 |
| NPC9 | 42 | 39 | 5 | 6 | 1 |

Illness Severity was determined by the disease disability rating scale developed by Itturiaga et al. (2006).

Psychosis Status was determined clinically by the presence of delusions or hallucinations

| **Supplementary Table 3. Distributions of connectivity measures at a global level** | | | | | | |
| --- | --- | --- | --- | --- | --- | --- |
|  | **Group** | **N** | **Mean** | **Median** | **SD** | **SE** |
| **Total FBC** | HC | 70 | 6.93 x10^6^ | 6.99 x10^6^ | 542613.2 | 64854.68 |
|  | NPC | 9 | 5.55 x10^6^ | 5.28 x10^6^ | 848058.8 | 282686.3 |
| **Average streamline length** | HC | 70 | 73.78 | 73.411 | 3.2554 | 0.38909 |
|  | NPC | 9 | 67.824 | 68.36 | 4.4651 | 1.48837 |
| **Total streamline count** | HC | 70 | 3.98 x10^6^ | 4.00 x10^6^ | 261444.8 | 31248.63 |
|  | NPC | 9 | 3.35 x10^6^ | 3308599 | 387426.8 | 129142.3 |
| **Mean FA** | HC | 70 | 0.348 | 0.348 | 0.0215 | 0.00257 |
|  | NPC | 9 | 0.277 | 0.273 | 0.0527 | 0.0176 |

| **Supplementary Table 4. Sex differences in connectivity measures of NPC patients** | | | | | | | | |
| --- | --- | --- | --- | --- | --- | --- | --- | --- |
|  |  | **Statistic** | **df** | **p** | **Mean difference** | **SE difference** | | **Effect Size** |
| **Total FBC** | **Student's t** | 1.491 | 7 | 0.18 | 789971 | 529835.4 | Cohen's d | 1 |
| **Mean FA** | **Student's t** | 0.99 | 7 | 0.355 | 0.035 | 0.0354 | Cohen's d | 0.664 |
| **Average length** | **Student's t** | -1.485 | 7 | 0.181 | -4.1461 | 2.7924 | Cohen's d | -0.996 |
| **Total streamline count** | **Student's t** | 1.823 | 7 | 0.111 | 417084 | 228785.4 | Cohen's d | 1.223 |

| **Supplementary Table 5. Significant edges, ranked by test statistic, with corresponding percentage change in FBC, FA, streamline count, length and volume for each connection.** | | | | | | |
| --- | --- | --- | --- | --- | --- | --- |
| **Node 1** | **Node 2** | **Test stat** | **% change FBC** | **% change**  **streamline count** | **% change**  **mean FA** | **% change streamline length** |
| R Thalamus | R Hippocampus. | 6.17 | -45.28 | -49.91 | -11.76 | -20.04 |
| L precentral | L Putamen. | 6 | -37.43 | -24.02 | -2.44 | -8.21 |
| R Putamen | R precentral. | 5.86 | -38.38 | -22.32 | -5.00 | -8.09 |
| L middletemporal | L superiorfrontal. | 5.66 | -79.60 | -72.62 | -9.52 | -4.54 |
| R Pallidum | R precentral. | 5.55 | -59.41 | -38.62 | -4.44 | -5.76 |
| L postcentral | L Putamen. | 5.53 | -33.77 | -28.51 | -4.88 | -8.00 |
| L inferiortemporal | L superiorfrontal. | 5.46 | -86.25 | -79.99 | -9.52 | -1.95 |
| R Putamen | R postcentral. | 5.35 | -41.09 | -35.72 | -5.00 | -9.54 |
| L superiorfrontal | L Thalamus. | 5.3 | -38.52 | -7.28 | -4.76 | -11.54 |
| L middletemporal | R superiorfrontal. | 5.25 | -85.60 | -84.11 | -17.78 | -19.56 |
| L superiorfrontal | L Cerebellum-Cortex. | 5.2 | -55.90 | -48.81 | -4.55 | -1.92 |
| R Pallidum | R postcentral. | 5.12 | -66.10 | -54.45 | -6.82 | -0.96 |
| R Putamen | R lateraloccipital. | 5.1 | -56.52 | -57.99 | -9.30 | -2.44 |
| L lateraloccipital | L Putamen. | 5.08 | -60.96 | -61.92 | -11.11 | -3.18 |
| L caudalmiddlefrontal | R superiorfrontal. | 5.08 | -54.37 | -56.17 | -8.51 | -3.63 |
| L precentral | L Pallidum. | 4.96 | -47.12 | -29.81 | 0.00 | -3.85 |
| L precentral | R paracentral. | 4.84 | -62.06 | -66.22 | -8.16 | -0.52 |
| L inferiortemporal | L Thalamus. | 4.82 | -69.98 | -65.45 | -7.69 | -3.21 |
| L lingual | L Putamen. | 4.68 | -66.69 | -68.95 | -10.87 | -5.75 |
| L middletemporal | L parsopercularis. | 4.67 | -69.52 | -64.22 | -11.36 | -5.36 |
| L superiorfrontal | R precuneus. | 4.66 | -80.21 | -81.01 | -12.50 | 2.47 |
| L paracentral | R paracentral. | 4.58 | -64.80 | -66.65 | -10.20 | -2.09 |
| L paracentral | R precentral. | 4.56 | -52.87 | -53.82 | -8.16 | -3.18 |
| L postcentral | L Cerebellum-Cortex. | 4.55 | -54.55 | -52.83 | -2.22 | -0.75 |
| L precentral | L Thalamus. | 4.53 | -36.40 | -11.33 | 2.33 | -5.59 |
| L Cerebellum-Cortex | L Putamen. | 4.51 | -49.44 | -51.17 | -4.88 | -1.05 |
| R Caudate | R parsorbitalis. | 4.43 | -63.04 | -60.64 | -9.09 | -12.96 |
| L fusiform | L Putamen. | 4.42 | -62.14 | -62.24 | -9.52 | -1.32 |
| L parsopercularis | R superiorfrontal. | 4.42 | -64.99 | -68.94 | -10.87 | -3.93 |
| L rostralmiddlefrontal | R Cerebellum-Cortex. | 4.37 | -75.17 | -70.64 | -23.81 | -17.68 |

**Data Availability – Code**

DWI Preprocessing

dwidenoise [INPUT_DWI] [DWI_DENOISED] -noise [NOISE_MAP]

mrcalc [INPUT_DWI] [DWI_DENOISED] -subtract [RESIDUAL_MAP]

mrdegibbs [DWI_DENOISED] [DWI_DEGIBBS]

dwiextract [DWI_DEGIBBS] - -bzero | mrmath - mean [MEAN_B0] -axis 3

mrconvert [MEAN_B0] [B0_NIFTI]

mrconvert [T1_IMAGE] [T1_NIFTI]

#Create acquisition parameters file (acqparams.txt)

#Run Synb0-DisCo (Synthetic b0 for Distortion Correction)

docker run --rm \

-v [INPUT_DIR]:/INPUTS/ \

-v [OUTPUT_DIR]:/OUTPUTS/ \

-v [FREESURFER_LICENSE]:/extra/freesurfer/license.txt \

--user $(id -u):$(id -g) \

leonyichencai/synb0-disco:v3.1

dwibiascorrect ants [INPUT_DWI] [OUTPUT_DWI_UNBIAS]

dwi2mask [INPUT_DWI] [BRAIN_MASK]

maskfilter -npass 2 [BRAIN_MASK] dilate [DILATED_MASK]

Prepare Index File for Eddy

mrconvert [INPUT_DWI] [DWI_NIFTI]

fslinfo [DWI_NIFTI] | awk 'FNR == 5 {print $2}' > [DIM4_FILE]

# Create index file with all 1s for single acquisition

for ((j=1; j<=[DIM4]; j++)); do echo -n "1 "; done > [INDEX_FILE]

Eddy Current and Motion Correction

mrconvert [DWI_DEGIBBS] [DWI_NIFTI] -export_grad_fsl [BVECS] [BVALS]

dwiextract [DWI_DEGIBBS] - -bzero | mrmath - mean [B0_EDDY] -axis 3

mrconvert [B0_EDDY] [B0_EDDY_NIFTI]

bet [B0_EDDY_NIFTI] [BRAIN_MASK] -m -f 0.1 -g -0.1

eddy --imain=[DWI_NIFTI] \

--mask=[BRAIN_MASK] \

--acqp=[ACQPARAMS] \

--index=[INDEX_FILE] \

--bvecs=[BVECS] \

--bvals=[BVALS] \

--topup=[TOPUP_OUTPUTS] \

--out=[DWI_CORRECTED]

T1 Processing and Registration

fsl_anat -i [T1_IMAGE] -o [T1_OUTPUT]

epi_reg --epi=[DWI_B0] --t1=[T1_BIASCORR] --t1brain=[T1_BRAIN] --out=[EPIREG_OUTPUT]

transformconvert [TRANSFORM_MAT] [DWI] [T1] flirt_import [MRTRIX_TRANSFORM]

mrtransform [T1] -linear [MRTRIX_TRANSFORM] -inverse [T1_REGISTERED]

Tissue Segmentation

5ttgen fsl [T1_REGISTERED] [5TT_IMAGE] -premasked

Response Function and CSD

dwi2response dhollander [DWI] [WM_RESPONSE] [GM_RESPONSE] [CSF_RESPONSE]

ss3t_csd_beta1 [DWI] [WM_RESPONSE] [WM_FOD] [GM_RESPONSE] [GM_FOD] [CSF_RESPONSE] [CSF_FOD] -mask [MASK]

mtnormalise [WM_FOD] [WM_FOD_NORM] [GM_FOD] [GM_FOD_NORM] [CSF_FOD] [CSF_FOD_NORM] -mask [MASK]

Tractography

5tt2gmwmi [5TT_IMAGE] [GM_WM_SEED]

tckgen -act [5TT_IMAGE] -seed_gmwmi [GM_WM_SEED] -maxlength 250 -seeds 25000000 [WM_FOD_NORM] [TRACTOGRAM]

tcksift2 -act [5TT_IMAGE] [TRACTOGRAM] [WM_FOD_NORM] [SIFT_WEIGHTS]

Atlas Registration

labelconvert [FREESURFER_PARC] [FREESURFER_LUT] [MRTRIX_LUT] [ATLAS_NOREG]

mrtransform [ATLAS_NOREG] -linear [TRANSFORM] -inverse -datatype uint32 [ATLAS_REGISTERED]

Connectome Construction

#SIFT2-weighted streamline density

tck2connectome -symmetric -zero_diagonal -tck_weights_in [SIFT_WEIGHTS] [TRACTOGRAM] [ATLAS] [CONNECTOME_SIFT2]

#Streamline count

tck2connectome -symmetric -zero_diagonal [TRACTOGRAM] [ATLAS] [CONNECTOME_COUNT]

#FA-weighted connections

tcksample [TRACTOGRAM] [FA_IMAGE] [FA_PER_STREAMLINE] -stat_tck mean

tck2connectome -symmetric -zero_diagonal -scale_file [FA_PER_STREAMLINE] -stat_edge mean [TRACTOGRAM] [ATLAS] [CONNECTOME_FA]

#Mean streamline length

tck2connectome -symmetric -zero_diagonal -scale_length -stat_edge mean [TRACTOGRAM] [ATLAS] [CONNECTOME_LENGTH]

**Reference**

1. Iturriaga, C., M. Pineda, E.M. Fernández-Valero, M.T. Vanier, and M.J. Coll. “Niemann–Pick C Disease in Spain: Clinical Spectrum and Development of a Disability Scale.” *Journal of the Neurological Sciences* 249, no. 1 (2006): 1–6. <https://doi.org/10.1016/j.jns.2006.05.054>.
